# Improving outcomes for patients discharged early using a home assessment scheme

**DOI:** 10.1080/17571472.2018.1489467

**Published:** 2018-07-09

**Authors:** Lucy Meehan, Ricky Banarsee, Val Dunn-Toroosian, Shafeeq Tejani, Alireza Yazdi

**Affiliations:** a Department of Academic Primary Care and Public Health, Imperial College London, Charing Cross Hospital, London, UK; b Clinical Research Network (CRN), National Institute of Health Research, North West London, UK; c NHS Brent Clinical Commissioning Group, Wembley Centre for Health & Care, London, UK; d Department of Medicine, Northwick Park Hospital, Harrow, UK

**Keywords:** Elderly-care, delayed discharge, patient experience, community

## Abstract

**Background:**

With increased delayed discharges from acute NHS hospitals, especially for older patients, solutions like the ‘Discharge to Assess’ (D2A) scheme aim to facilitate quicker discharge and improve experiences for patients and carers.

**Setting:**

This report examines the quality process from the patient perspective of the D2A scheme implemented in a London Northwest Healthcare NHS Trust (LNWHT). A retrospective audit was conducted using the first cohort of patients discharged through this pilot scheme from April to July 2017.

**Question:**

A brief study to explore patient views of their experience of the D2A scheme.

**Methods:**

An opportunistic audit comprised of brief telephone interviews with patients following discharge from hospital through the D2A scheme.

**Results:**

30 patients who had been discharged with the D2A scheme, agreed to participate. Overall, patients were positive about their experience and valued the support and services provided. However, there were concerns on the issue of communication. The scheme effectiveness from the patient’s perspective improved over the duration of the evaluation.

**Discussion:**

Patients’ views about their experiences changed over time, which included patients’ perceptions of the discharge process, patients’ expectations and the way in which they were able to access services.

## Why this matters to me

The population in the UK is ageing. The percentage of people aged 65 years and over is estimated to grow to nearly a quarter of the population by 2045 [3]. With ageing brings increased likelihood of having multiple long-term conditions and frailty. Frailty is recognised as a ‘state of vulnerability to adverse outcomes’ and consequently increases the chances of decompensation leading to hospital admission [4]. Frail patients have less physiological reserve meaning they do not recover as quickly from illnesses and require more time to facilitate recovery and more unlikely to return to baseline [4]. Their stay in hospital once ‘medically fit’ should not be increased further by awaiting assessments for social care needs, as this risks them to poorer health outcomes such as hospital acquired infections and sarcopenia [1,4].

Schemes that enable quicker discharge from hospital with social care assessments and rehabilitation support provided in the community hope to improve outcomes for patients individually and to more widely reduce bed pressures and financial strain on hospitals. As I train to be a GP and as the NHS changes to accommodate our aging population and manage more patients in the community, schemes such as Discharge to Assess are warmly welcomed to offer a framework for healthcare systems to improve the discharge process and experiences for patients [2].

Evaluating the D2A scheme through patient experience aims to provide insightful feedback of what works and what can be improved for patients as they are discharged from hospital and supported at home more quickly.

## Key message

Continuous quality improvement of the D2A scheme as it became more established.

## Introduction and background

In 2003 the Department of Health published a ‘Hospital Discharge Workbook,’ emphasising that discharge from hospital was not an isolated event but required a ‘whole systems’ approach [5]. The key principles identified for discharge included: active engagement of patients and carers, multidisciplinary working, effective communication between secondary and primary care, planning for discharge from admission and continued assessment of needs during a period of rehabilitation before any permanent decisions were made [5].

Difficulties in the discharge of older patients from NHS hospitals, continues to be an important issue, highlighted by the National Audit Office findings in May 2016 [1]. Between 2013 and 2015, 1.15 million hospital bed days were occupied by patients no longer in need of acute treatment, translating to an estimated NHS spend of £820 million per year on older patients who no longer needed to be in hospital [1,2]. In addition to the increased financial strain for trusts and ‘bed-blocking’ associated with delayed discharges, prolonged hospital admissions for older patients leads to poorer health outcomes and increase in their long-term care needs [1,2]. Older patients are at increased risk of hospital-acquired infections and risk decline in their mobility and independence with 5% loss in muscle strength with each day in hospital [1].

A relatively new scheme, ‘Discharge to Assess’ (D2A) aims to address the issues associated with delayed discharges and improve patient and carer experience [2]. Under this scheme, once patients are identified as ‘clinically optimised’, but still require support for care needs, they are discharged home or to another community setting [2]. Assessment for the patient’s care and support needs are then conducted in the patient’s own familiar environment, giving a better measure of what support they actually need [2].

The ‘D2A’ model aims to support patients to be discharged safely, with prompt assessment (within 2 hours of discharge) and rapid (on the day) access to care and support if required [2]. The support services offered with D2A should be time limited (up to 6 weeks), and assessment of longer-term care needs can then be more accurately assessed [2]. The D2A scheme also aims to place patients and carers at the centre of decisions regarding their care [2].

## Setting

The service evaluated was a ‘Discharge to Assess’ scheme, (now re-named ‘Home First’), initiated in an area of Northwest London in April 2017.

## Objectives

The main objectives were to explore the patient’s experiences of hospital discharge under the D2A scheme by exploring whether patients and carers were involved and informed about discharge planning and assessments, their experiences of the D2A process and experiences of the support provided in the community setting and whether their care needs were being met in the community.

## Methodology

This was a qualitative study comprising of a retrospective audit evaluating patient experiences discharged through the D2A scheme.

**Figure 1.**
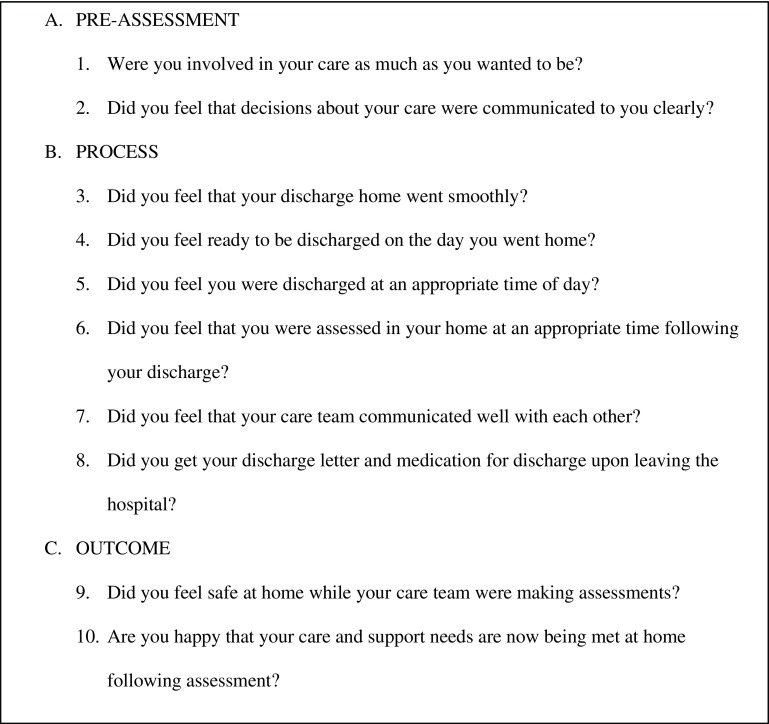
Patient survey protocol.

The data were collected via informal telephone interviews with patients and/or carers using a questionnaire protocol of 10 questions (see Figure 1) and focused on key elements of the D2A process:

(a) Experience of pre-assessment
(b) Experience of the process (discharge and assessments)
(c) Outcomes: were the patients/carers satisfied with the care and support in meeting their needs

A convenient sample was identified as the first cohort of patients discharged through D2A from April to July 2017 in an area of Northwest London. The patients selected for interview were based on a ‘28-day criteria’ where any patients re-admitted within 28 days from discharge were considered a ‘failure’ of the scheme and not included. One-to-one telephone interviews were conducted in August and September 2017.

Respondents were encouraged to speak in their own words giving as much detail as they wished. Interviewers encouraged elaboration when necessary, using the protocol to standardise the interviews. The telephone interviews were manually recorded and all data collected made anonymous.

## Results

63 patients were identified in the cohort of patients discharged with the D2A scheme from April to July 2017 that met the above criteria of the ‘28 day rule.’ However when contacted for the interview, 2 patients had died and 4 had been re-admitted to hospital within 28 days. Of the remaining patients, 52% of patients (*n* = 30) were successfully contacted and agreed to participate in the telephone interviews.

Overall, of the majority of patients and carers contacted, 60% positively rated the scheme. They welcomed the D2A scheme as a new model where assessment took place at home rather than in hospital in order to facilitate their discharge sooner.

The data revealed a trend - dissatisfaction was higher in the first months and steadily improved. See Table 1.

**Table 1.** Patients’ views of service delivery.

Month	June	July	August	Average
Positive	5 (50%)	6 (60%)	7 (70%)	18 ≫ 60%
Negative	5 (50%)	4 (40%)	3 (30%)	12 ≫ 40%

### Trend analysis

Although on aggregate, the ratio of positive versus negative perception was around 60:40, when individual questions were scrutinised, a slightly different picture emerged. In the first month of the scheme, perception of service was at its lowest. This improved over time. By month 4 (August) of the scheme in operation, it was more positively perceived (70%).

See Table 2 for a breakdown of percentage negative and positive responses for the individual protocol questions.

**Table 2.** List of individual discharge protocol questions.

	Month	June		July		August	
	Response – *P* (positive)/*N* (Negative) – %	*P* (+) - %	*N*(–) – %	*P*(+) – %	*N*(-) - %	P(+) – %	*N*(–) – %
	*N* = Subjects interviewed	10		10		10	
	
Pre-Assessment	I was involved in my care as much as I wanted to be	55	45	60	40	75	25
	I felt that decisions about my care were communicated to me clearly	55	45	60	40	65	35
Process	I felt that my discharge home went smoothly	50	50	60	40	65	35
	I felt ready to be discharged on the day you went home	55	45	70	30	70	30
	I felt I was discharged at an appropriate time of day	55	45	60	40	80	20
	I felt I was assessed in my home at an appropriate time following your discharge	55	45	55	45	75	25
	I felt that my care team communicated well with each other	40	60	55	45	60	40
	I got my discharge letter and medication for discharge upon leaving the hospital	35	65	60	40	65	35
Outcomes	I felt at home while your care team were making assessments	50	50	60	40	75	25
	I feel happy that my care and support needs are now being met at home following assessment	50	50	60	40	70	30
							
	Average response (per period)	50	50	60	40	70	30

#### A. Pre-assessment

Being part of the process

**Aim:**

Patients and carers must to be involved in the discharge process from the outset.

**Findings:**

Whilst generally positive, some patients and carers felt they were ignored. In the patients and carers who cited negative experiences with the D2A scheme, communication was the most common issue raised. 40% of patients felt that they were not adequately consulted about decisions regarding their care and 35% of carers felt that they were often omitted from decision-making but felt that their input was important.

**Comments**

Very satisfied. Everything was explained to me and I have no problem at all. (Interview 1 - patient)

No one asked my opinion. As if I was not there. (Interview 4 - carer)

The doctors and nurses were very, very kind. They explained in detail what was going to happen to my husband. (Interview 12 - patient)

#### B. Process of care and assessments in the community

1. Timing and delivery

**Aim:**

The scheme initially promised that all assessments would be within two hours of being discharged. That was difficult to audit, as there was no available data. Future evaluation will need to re-visit this.

**Findings:**

All patients were asked to describe, in their own words, how they felt about being discharged. Although around 60% (*n* = 18) felt positive about their discharge, 12 felt strongly that there were problems regarding follow up services discussed in their plans.

**Comments**

I was glad I came home. I just didn’t like being in the hospital. The nurses were really nice. (Interview 7 - patient)

I had a stoma and need intense physiotherapy twice a week, I’ve had only 2 visits in the last 2 weeks. (Interview 4 - patient)

I thought they would have contacted my GP. He did not seem to know anything. (Interview 16 - patient)

2. Communication

40% of participants felt that their communication around needs assessments and discharge planning was poor. Specific issues highlighted regarding communication were that some patients returned home with no information about self-care or actions to take should complications or relapses occur, and some commented that they returned home with no information about local support agencies.

Other specific issues relating to the process included: follow up not materialising (for example physiotherapy), some patients had difficulty with everyday tasks such as walking and shopping, and some felt the needs assessments were carried out in an unrealistic way so were not accurate.

3. Documentation

Patients were appreciative that the doctors and nurses took the time and trouble to give them more general information about their condition and management. However, for many they do not recall or have evidence of discharge summaries. This was difficult to verify from the telephone interviews.

#### C. Outcomes

Whilst most were complimentary to the service, there were some patients who used words like ‘angry,’ ‘disgusted,’ and ‘abandoned’ to describe their feelings. Our analysis focused on seeking to understand why patients felt the way they did about discharge. Apart from communication, it was about what the patients believe the scheme was about. For some it was confusing and disorganised.

**Figure 2.**
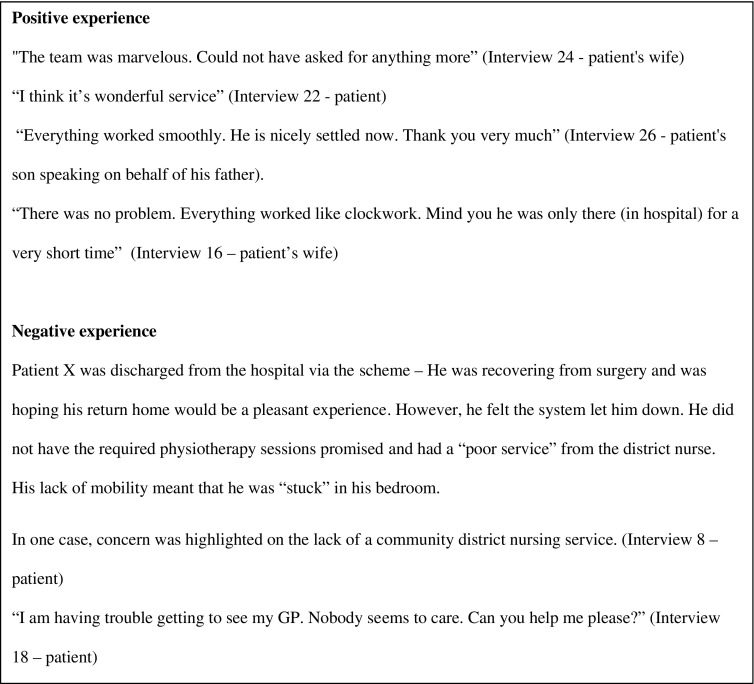
Some patient and carer comments from the interviews.

Figure 2 displays a selection of positive and negative comments quoted by the patients and carers when interviewed.

## Limitations

The sample size was small, with a response rate of 52%. The results from this local study should be replicated over a longer time to capture more patient experiences.

The audit questionnaire was retrospective and for some patients the telephone interview occurred weeks to months after their experience with the D2A scheme. For patients who may have memory difficulties or who have had more than one hospital admission, they may have difficulty in remembering their experience of the D2A scheme. This could be minimised by prospectively capturing data by giving patients and carers a questionnaire at the time of discharge and at the end of the D2A process provided in the community.

The study focused on patient experience as a measure of the D2A process. This cannot be standardised as it is based on individual patient opinion and their perception of the same team or process will vary person to person. For example, aspects of a process which may bother a certain patient such as time of discharge may be more suitable for one patient but unsuitable for another. More thorough evaluation of the D2A process would be achieved by also evaluating the professionals’ experience of it. The quality of the study could also be improved by adding quantitative measures such as economic impact to provide evidence about its cost effectiveness of D2A.

## Conclusion and Discussions

Evidence from this audit suggests that the introduction of the D2A scheme was generally well received and had improved outcomes for some patients. Although there were negative aspects highlighted, no formal complaints were received by either health or social care during the study period.

Interestingly, the results demonstrated a trend whereby patient’s opinion and experience of the D2A scheme became more positive in the later months. This may be explained by the D2A process becoming more established and familiar to all concerned as time progressed.

Regarding the negative response highlighted, communication surrounding discharge process and care assessments for patients needs to be improved. A specific recommendation to enhance communication and to ensure a patient centred approach could be to provide an information pack for patients and carers. This information pack would outline the D2A scheme, including the discharge process and assessment of care and support needs, contacts for the MDT professionals involved, support available in the community for that patient and a personalised record of discussions and decisions made with the patient and carers. This pack would then be available for the patient and multidisciplinary team members of the D2A teams to enhance continuity. The hospital discharge summary and instructions for self-care and what to do if things don’t go as expected could also be included in this pack.

The D2A scheme has been shown to be a positive experience for the majority of participants interviewed and meets the D2A objective to aid early effective discharge from hospital. This, although not confirmed with this study, should lead to positive outcomes for the NHS and for the longer-term health and wellbeing of patients individually.

## Governance

Ethical approval was not deemed necessary as the audit was anonymous and was auditing the service provision of a new discharge scheme.

## Disclosure statement

No potential conflict of interest was reported by the authors.

## Related LJPC Papers

The integrated care pilot in north west London, Dec 2012, LJPC 2012;5:8-11
